# The neuropeptide Y receptor gene repository, phylogeny and comparative expression in allotetraploid common carp

**DOI:** 10.1038/s41598-022-13587-2

**Published:** 2022-06-08

**Authors:** Xiaoqing Zou, Lin Chen, Bijun Li, Junzhu Xiao, Peng Xu

**Affiliations:** 1 State Key Laboratory of Marine Environmental Science, College of Ocean and Earth Sciences, Xiamen University, Xiamen, China; 2 Fujian Key Laboratory of Genetics and Breeding of Marine Organisms, College of Ocean and Earth Sciences, Xiamen University, Xiamen, China; 3 State Key Laboratory of Large Yellow Croaker Breeding, Ningde Fufa Fisheries Company Limited, Ningde, China

**Keywords:** Evolution, Molecular evolution, Ichthyology

## Abstract

NPY-family receptors belong to G protein-coupled receptors (GPCR), which lays a physiological foundation for the transmembrane transport of an endogenous appetite-stimulating factor neuropeptide Y and related peptides. In this study, we investigated the *npyr* genes in ten representative species, and twelve *npyr* genes were identified from allotetraploid *C. carpio*, the *npyr* gene number of *C. carpio* was twice the number of its subgenome B progenitor-like diploid *Poropuntius huangchuchieni*. Phylogenetic analysis showed that all *npyr* genes were divided into three subgroups, and they underwent strong purifying selection according to selection pressure analysis. Subsequently, synteny analysis showed that most *npyr* genes were evenly distributed on the homologous chromosomes of two subgenomes in allotetraploid *C. carpio*, in which *npy1r* and *npy2r* were tandem duplicated, respectively. In addition, the global expression of *npyr* genes during embryonic development in allotetraploid *C. carpio* suggested the potential function of *npyr* genes in immunity and reproduction. In adult tissues, *npyr* genes were mainly distributed in the brain, gonad, and skin, which displayed a similar expression pattern between the *C. carpio* B subgenome and *P. huangchuchieni*. In general, our research could provide reference information for future exploration of the NPY receptors and neuroendocrine system of allotetraploid *C. carpio* and vertebrates.

## Introduction

Neuropeptide Y (NPY) and its related peptides usually affect the neuroendocrine system of organisms by acting on several G protein-coupled receptors^1^, including the regulation of appetite^2^ and response to anxiety^3^ as well as surrounding pressure^4^, and act as immune roles^5^. NPY-family receptors are thought to be mainly expressed in fish brains, as well as in marginal tissues such as eyes and intestines^6^. Based on the structural characteristics of the receptor and the amino acid sequence similarity, NPY-family receptors are generally divided into seven subtypes: Y1, Y2, Y4, Y5, Y6, Y7 and Y8^6^. They all belong to the G protein-coupled receptors. Among them, Y1, Y4, Y6, Y8a and Y8b belong to the subfamily of Y1 NPY-family receptor genes^7^. The fifth subtype Y6 usually exists in primates in the form of pseudogenes^8^. There is only one subtype in the Y5 category and it does not have an additional copy. Y2 and Y7 both belong to the Y2 subtype, which is due to the increased copy of gene replication^9,10^. All members of the NPY-family receptor are thought to have originated from a single receptor gene in the ancestors of all vertebrates through multiple rounds of whole-genome duplication^8^.

It has been reported that Y1, Y2, Y4, Y7, Y8a and Y8b are all present in teleost^9^. The Y1-like and Y5 receptor genes were found in basal teleost^1,11^, while Y1 and Y5 were thought to be missing in both zebrafish and pufferfish^10,12^. The loss of these genes may be a consequence of large-scale duplication in the process of lineage evolution^10^. But the Y1 receptor was identified in whole-genome data of zebrafish later^13^. Y1 receptors are considered to be receptors for promoting food intake, and studies in goldfish have shown that the effect of appetite-stimulating factors on food intake is partly mediated by Y1 receptors rather than Y2 receptors^14^. The Y2 receptor has attracted much attention as an appetite suppressor receptor expressed in the hypothalamic arcuate nucleus^15^, and the Y7 receptor is considered to be a direct homologue of Y2^8^. The pharmacological properties of Y7 were studied in zebrafish, and it was found that the Y7 receptor was more sensitive to the truncation of amino terminals of peptide ligands than Y2^16^. Both Yb and Yc, which were first identified in zebrafish, were identified as Y8a and Y8b later, and the two receptor genes were also identified in pufferfish. Their chromosome location showed that they were most likely to occur in teleost whole-genome duplication (3R)^10,17^.

The evolutionary dynamics of NPY and its receptors in whole-genome duplication events in early vertebrates have been thoroughly studied^1,8^. Similarly, the fate of NPY and its receptor genes in the third round of teleost-specific whole-genome duplication events (*Ts3R* WGD) have been studied thoroughly^13,17^. The NPY-family receptors of autotetraploid fish like salmon and trout have been identified, but the duplicated Y2 and Y7 receptors may not be found due to the incompleteness of the genomic DNA library^12^. The occurrence of autotetraploidy events in salmon and trout is relatively early^18^ and rediploidization and gene loss may have happened^19^. The evolutionary fate of NPY-family receptor genes in common carp that experienced the most recent polyploidy is still unknown.

As a type of polyploid fish widely distributed worldwide, common carp have strong stress resistance^20–22^. According to the Ka/Ks analysis of homologous genes and the divergence rate of transposable elements, it is inferred that the tetraploidy event of common carp may have occurred 12.4 million years ago (Mya) and coincided with the climate change event driven by the sharp uplift in the eastern Qinghai-Xizang Plateau^23^. The allotetraploid common carp can be divided into two sets of subgenome A and B according to the coverage of each of the 50 pairs of chromosomes between the related species^23^. Based on systematic genomic evidence, it has been determined that Poropuntius–Puntius–Hampala is a closely related diploid progenitor-like group of common carp B subgenome^23^, and representative diploid *Poropuntius huangchuchieni* and *Onychostoma macrolepis* were included in this study.

This study led to a better understanding of the evolutionary fate of *npyr* genes in allotetraploid common carp after the fourth round of whole-genome duplication events. The abundance and phylogeny relationships of *npyr* genes were analyzed in ten species, as well as allotetraploid common carp and related diploid and autotetraploid species. Structure and conservative motif analysis provide evidence for the classification of *npyr* genes. Based on the results above, we also conducted a comparative expression analysis of *npyr* genes in allotetraploid common carp and related diploid species. The abundance, distribution and expression profile of *npyr* genes in allotetraploid common carp provide us with more comprehensive information about the evolutionary footprint and functional exploration of *npyr* genes after the fourth round of whole-genome duplication event.

## Materials and methods

### Identification and nomenclature of *npyr* genes

In order to identify the *npyr* genes in the whole-genome of allotetraploid *C. carpio*^23^ and its subgenome B progenitor-like diploid *P. huangchuchieni*^24^, we used the sequences of *npyr* genes such as Y1, Y2, Y4, Y7and Y8a in *Danio rerio*, which were downloaded from Ensembl (https://uswest.ensembl.org/index.html) as queries. Both BLASTP and tBLASTn were used to search against newly published genomes for comprehensive investigations of *npyr* genes. The other seven species investigated in this study include *Homo sapiens*, diploid fish *Oryzias latipes*, *Poropuntius huangchuchieni*, *Onychostoma macrolepis*, *Takifugu rubripes* and tetraploid fishes *Carassius auratus*, *Salmon salar* and *Oncorhynchus mykiss*. The acronym was used to replace the Latin name of the species. For example, *Homo sapiens* was abbreviated as *Hsa* or *H. sapiens*. All *npyr* genes in *C. carpio* and other species were renamed according to its *D. rerio* ortholog (*npy1r* and *npy2r*, etc.), with species abbreviated as a prefix and numbered suffixes (*Cca_npy1r-1*, *Cca_npy1r-2*, etc.).

### Phylogenetic analysis

Multiple sequence alignment was conducted by clustalx2.1^25^ and visualized by ESPript (https://espript.ibcp.fr/ESPript/cgi-bin/ESPript.cgi)^26^. All the NPY receptor sequences of ten species were aligned via MEGA X^27^ built-in alignment button MUSCLE^28^ and then manually trimmed. We performed the phylogenetic analysis with RA × ML (version 8.2.12) and obtained the maximum likelihood (ML) tree. Subsequently, the online tool EvoView (https://evolgenius.info/evolview-v2/#login)^29^ was used to visualize the phylogenetic tree, the same method was used in producing species trees based on their shared *npy2r* genes.

### Structure and conservative motif analysis

We used a simple modular architecture research tool (SMART http://smart.embl-heidelberg.de/) to predict conserved domain architectures of NPY receptors in *C. carpio*. The results were visualized by IBS (version 1.0). In addition, motif analysis was performed by using MEME (https://meme-suite.org/meme/tools/meme)^30^, including the motif logo specific to different subtypes of NPY receptors were visualized through TBtools (v1.0695)^31^. Additionally, the protein structure of NPY receptors in *C. carpio* was predicted and represented by Protter^32^.

### Calculation of selective pressure

We used the evolutionary ratio of non-synonymous substitutions (dN) and synonymous substitutions (dS)^33^ on the NPY-family receptor gene sites to characterize the selection pressure. The complete amino acid sequences of Y1, Y2, Y4, Y7, Y8a and Y8b receptors were aligned with the MUSCLE in MEGA X to eliminate terminators. Then we calculated the selection pressure of each site at the codon level through the Single Likelihood Ancestor Count (SLAC) method on the Datamonkey server (http://www.datamonkey.org/)^34^.

### Synteny analysis and comparative expression profiles

To exhibit the syntenic relationship of the *npyr* genes in allotetraploid *C. carpio* and related diploid fishes, the syntenic plots were constructed with a multiple collinearity scan toolkit (MCScanX)^35^.

In order to compare the expression profiles of *npyr* genes in different tissues and embryo developmental stages in allotetraploid *C. carpio* and *C. carpio* B subgenome progenitor-like diploid *P. huangchuchieni*, we analyzed transcriptome datasets of 11 embryo developmental stages (Sperm, Egg, Zygote, Morula, Blastula, Gastrula, Neurula, Optic vesicle, Tail bud, Muscle contraction, 1dph) of *C. carpio* and 12 adult tissues of *C. carpio* and *P. huangchuchieni* using Hisat2^36^ and Stringtie^37^, all of these tissues were taken from the healthy and untreated fish and the TPM (Transcripts per million) value was used to qualify gene expression levels of NPY receptor coding genes. The transcriptome dataset of *C. carpio* was deposited in NCBI, with accession number PRJNA689982 (https://dataview.ncbi.nlm.nih.gov/object/), and the National Genomics Data Center under accession number PRJCA004216 (https://bigd.big.ac.cn/), and the transcriptome dataset of *P. huangchuchieni* was available at the National Genomics Center under Bioproject number PRJCA002855 (https://bigd.big.ac.cn/gsub/submit/bioproject/subPRO004206/overview). The visualization was presented by the dynamic heatmap module of an online heatmap tool (http://www.omicshare.com/tools/Home/Soft/heatmap).

### Ethics approval

Animal treatments in the study were conducted following the regulations of the Guide for Care and Use of Laboratory Animals and approved by the Committee of Laboratory Animal Experimentation at College of Ocean and Earth Sciences, Xiamen University.

## Results

### Genomic investigation of the *npyr* gene repertoire

A total of 12 *npyr* genes were identified in the common carp genome, including Y1, Y2, Y4, Y7, Y8a and Y8b, all of which have two copies, and were evenly distributed on the homologous chromosomes of two subgenomes (Table 1). The results showed that the length of the sequence of *npyr* genes varied from 370 to 381 amino acids (aa). Except for *npy1r-1* and *npy1r-2* which had two exons, all of the *npyr* genes in the common carp only had a single exon.

**Table 1:** Detailed information about neuropeptide Y receptor (*npyr*) gene in *C.carpio*.

Gene name	Chromosome location	Genomic length (bp)	CDs (aa)	No. of exons	Accession no
*npy1r-1*	A01	9783	380	2	OK180491
*npy1r-2*	B01	10,595	381	2	OK180492
*npy2r-1*	A01	1124	374	1	OK180493
*npy2r-2*	B01	1112	370	1	OK180494
*npy4r-1*	A17	1133	377	1	OK180495
*npy4r-2*	B17	1133	377	1	OK180496
*npy7r-1*	A14	1130	378	1	OK180497
*npy7r-2*	B14	1130	376	1	OK180498
*npy8ar-1*	A10	1133	377	1	OK180499
*npy8ar-2*	B10	1136	376	1	OK180500
*npy8br-1*	A08	1133	377	1	OK180501
*npy8br-2*	B08	1133	377	1	OK180502

**Figure 1:**
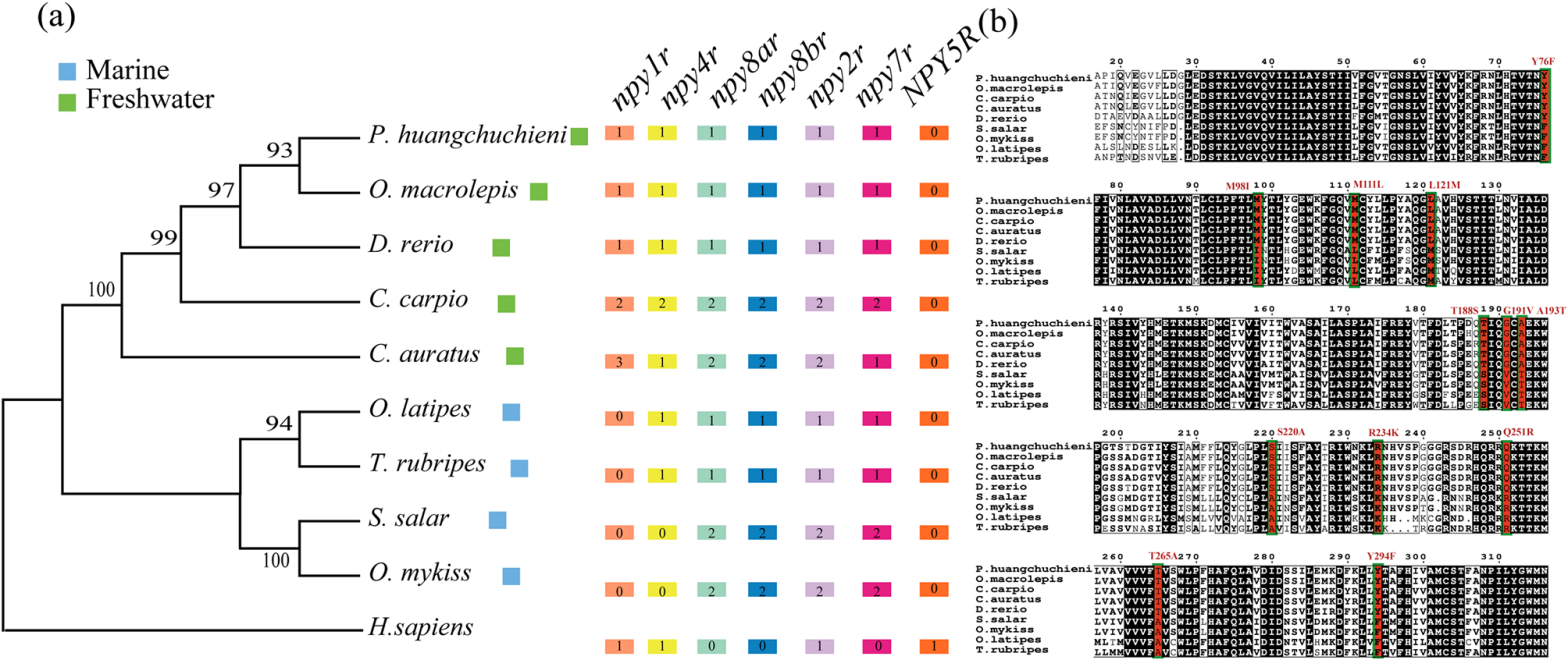
Comparison of gene copy numbers of *npyr* genes among selected vertebrate genomes from marine and freshwater habitats. (**a**) The phylogenetic tree of the species examined in this study was constructed using common Y2 receptor amino acid sequences. The blue rectangles represent marine fish, while the green ones represent freshwater fish. The colorful rectangles in the right panel are marked with the copy numbers of the *npyr* gene. (**b**) Amino acid sequence alignments of NPY receptors for all fish species examined. The conserved regions are indicated by a shadow. The black shadow indicates the region where all sequences share the same amino acid residue. The red bars indicate positions that vary between marine fish and freshwater fish. Species abbreviations include *H. sapiens*, *Homo sapiens*, *D. rerio*, *Danio rerio*, *S. salar*, *Salmo salar*, *O. mykiss*, *Oncorhynchus mykiss*, *O. latipes*, *Oryzias latipes*, *P. huangchuchieni*, *Poropuntius huangchuchieni*, *O. macrolepis*, *Onychostoma macrolepis*, *C. carpio*, *Cyprinus carpio*, *T. rubripes*, *Takifugu rubripes* and *C. auratus*, *Carassius auratus*.

The number of *npyr* genes in all investigated species is shown in Fig. 1a. The results showed that the number of *npyr* genes of allotetraploid common carp was twice as much as that in related diploid species, such as *D. rerio, O. macrolepis* and *P. huangchuchieni*, which indicated that the common carp had undergone the fourth round of whole-genome duplication event and that the *npyr* genes were duplicated in a whole-genome duplication event. However, the related allotetraploid goldfish Y1 receptor had three copies, whereas Y4 and Y7 had only one copy (Fig. 1a). This meant that the *npyr* genes in common carp and goldfish had a different fate after the fourth round of whole-genome duplication events.

As for autotetraploid, we found that the Y1 and Y4 receptors of *D. rerio* lacked orthologues in *S. salar* and *O. mykiss*, which have two copies of Y2, Y7, Y8a and Y8b. This showed that the doubling of *npyr* genes is indeed accompanied by a whole-genome duplication event. Regarding the loss of Y1 and Y4 receptors in two autotetraploids, we also searched for them in *O. latipes* and *T. rubripes*, and found that all marine fish (Fig. 1a) lack Y1 receptors compared with freshwater fish. The Y4 receptors are still retained in diploid species, but are missing in autotetraploids in marine habitats, which may be closely related to the early rediploidization event of autotetraploid^19^.

In order to confirm the characteristics of the *npyr* genes in teleosts from different habitats, we carried out multiple alignment of the *npy2r* amino acid sequences shared by teleosts in different habitats. All the sequence similarities were ranging from 70.25 to 95.92%, and the amino acid codes for pairs of conserved regions remained consistent with species of the same habitat, but there were exclusive site differences between different habitats. It was found that 12 residue sites were completely different between marine and freshwater habitats, for example, Y76F meant a mutation from Y to F at residue positions 76 among freshwater and marine habitats (Fig. 1b). We implied that these teleosts from two different habitats may have such possible species-specific mutation sites in completely conservative regions of the genome. These sites may be the main regulatory regions for the adaptive evolution of different habitats.

### Phylogenetic analysis of NPY-family receptor genes

**Figure 2:**
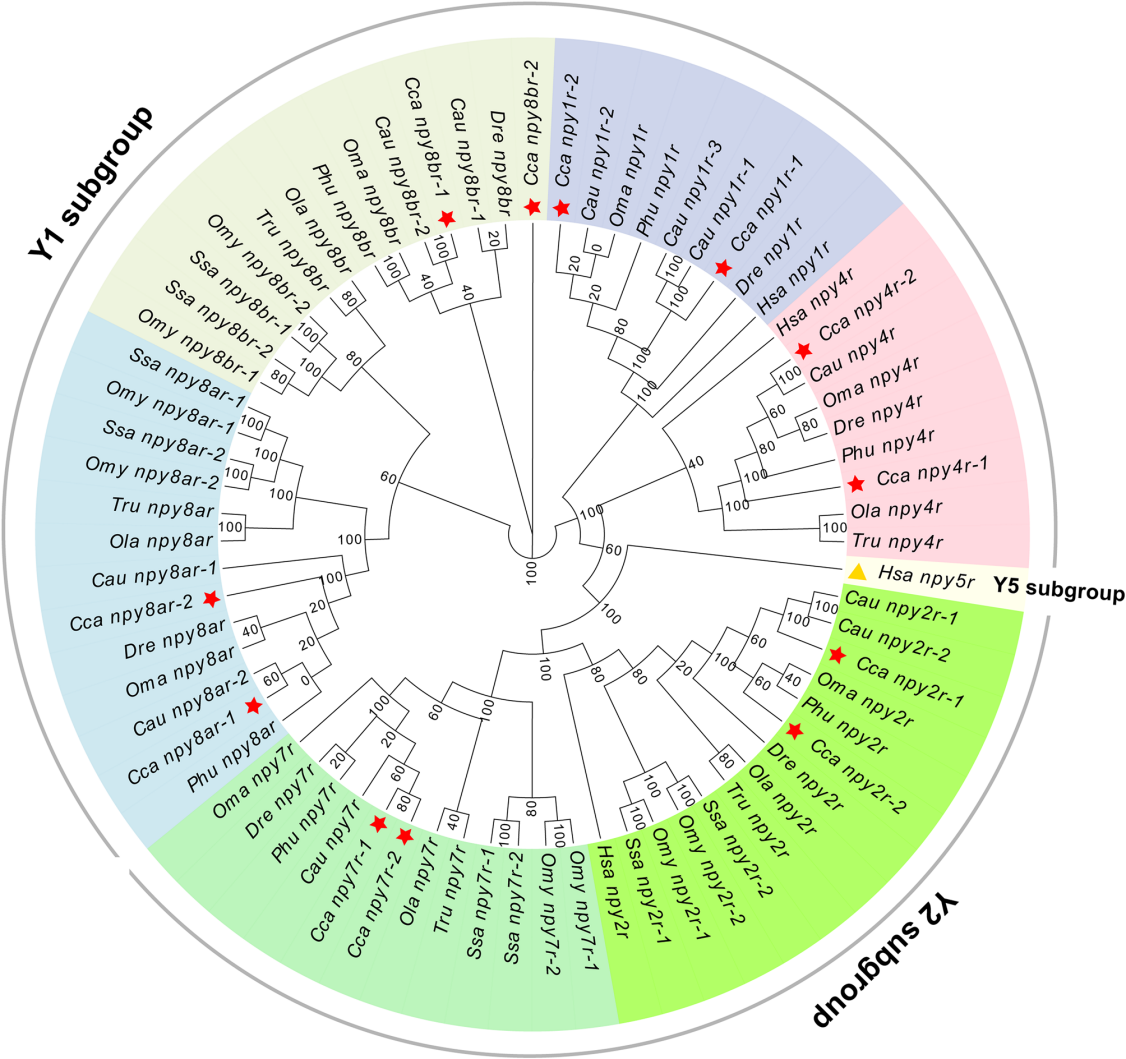
Phylogenetic tree of the NPY-family receptors in vertebrates. All of the same types of NPY-family receptors are shown to have the same color backgrounds as the NPY-family receptors of common carp that are marked by red stars. The one marked with a triangle is NPY5R, which is used as an outgroup of the phylogenetic tree. Abbreviations for species names: *Homo sapiens* (Hsa), *Danio rerio* (Dre), *Salmo salar* (Ssa), *Oncorhynchus mykiss*(Omy), *Oryzias latipes* (Ola), *Poropuntius huangchuchieni* (Phu), *Onychostoma macrolepis* (Oma), *Cyprinus carpio* (Cca), *Takifugu rubripes*(Tru), *Carassius auratus* (Cau).

To investigate the evolutionary relationship between the *npyr* genes in vertebrate lineages, we constructed a phylogenetic tree using 71 amino acid sequences of *npyr* genes in ten investigated species (Fig. 2). All *npyr* genes were divided into three subgroups, with Y1, Y4, Y8a and Y8b belonging to the Y1 subgroup that was clustered into one group, and Y2 and Y7 belonging to the Y2 subfamily were clustered into the other group. While NPY5R in humans was a separate group, it had no orthologs in fish (Fig. 2).

### Selection pressure of teleost *npyr* genes (dN/dS)

To obtain the evolutionary dynamics of *npyr* genes in the vertebrate evolutionary lineage, we conducted a selective pressure analysis and calculated the substitution rates of non-synonymous substitutions (dN) and synonymous substitutions (dS) (Table 2). The ratio of dN/dS below 1 indicates negative selection pressure (relaxed purifying selection), while a value above 1 indicates positive selection (selection for diversity), and dN/dS equal to 1 indicates neutral selection^38^. The dN/dS ratios of all groups in the survey appeared to be close to 0, which meant that NPY-family receptors have undergone pronounced negative selection in the vertebrate lineage, implying that these genes have essential functions on the growth and survival of organisms^39^.

**Table 2:** Selection pressure (dN/dS) of *npyr* genes in vertebrates (p  ≤ 0.05).

Gene	dN/dS	No. of positive sites	No. of negative sites
*npy1r*	0.120	0	20
*npy2r*	0.189	0	92
*npy4r*	0.180	0	33
*npy7r*	0.154	0	68
*npy8ar*	0.0916	0	91
*npy8br*	0.0988	0	75

### Structure analysis of the NPY receptors in allotetraploid common carp

NPY-family receptors belong to the G-protein coupled receptor and have seven transmembrane domains (7tm_1), which facilitate the transmembrane transport of NPY and related peptides, thereby acting on the body’s peripheral tissues, and regulating growth, food intake, immunity and other physiological functions^40^. According to the result of secondary structure prediction, we found that all *npyr* genes in allotetraploid common carp have seven transmembrane domains (Supplementary Fig. 1). In addition, low complexity sequences were found at the N-terminal of two copies of *npy4r*, all of which confirmed the accuracy of common carp *npyr* gene identification.

**Figure 3:**
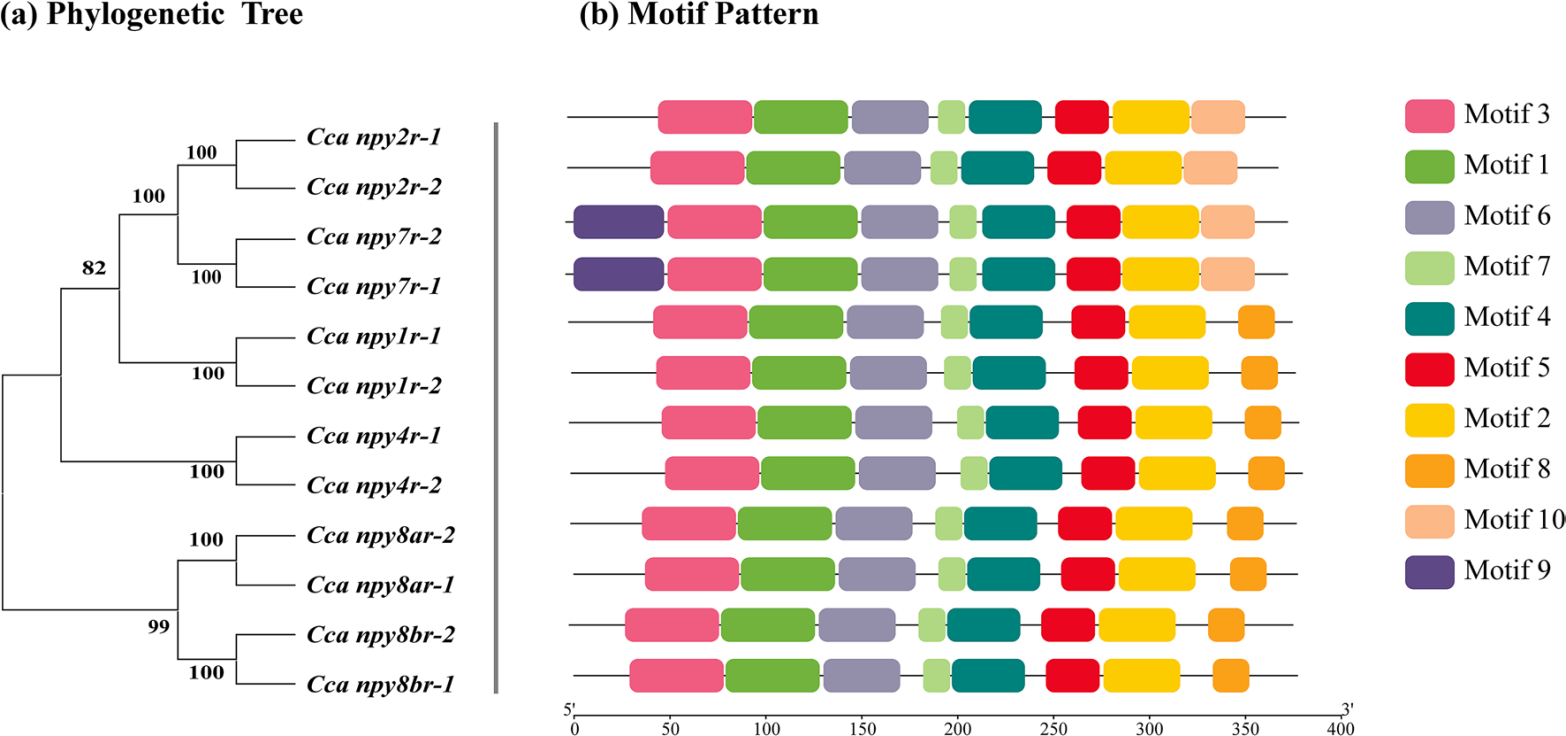
Phylogenetic tree and motif analysis of NPY-family receptor genes in allotetraploid *C.carpio*. (**a**) The phylogenetic tree of NPY-family receptor genes in allotetraploid *C. carpio*. (**b**) Sequences analysis of NPY-family receptors in *C.carpio*, with colored pieces representing conservative motif patterns on these receptors. Secondary structural analysis of these sequences is shown in supplementary Fig. 1.

The NPY-family receptors can be divided into three subfamilies based on amino acid sequence similarity and evolutionary origins, such as those of the Y1, Y2, and Y5 subgroups. The phylogeny trees of all *npyr* genes in allotetraploid common carp were constructed using their peptide sequences, showing the paralogous relationship of *npyr* gene copies and a clear cluster of two subtypes (Fig. 3a). Generally, the structure of the receptor protein is closely related to the affinity between receptor proteins and their related ligands. Motif analysis showed that most motifs of *npyr* genes in allotetraploid common carp are highly conservative (Fig. 3b).

**Figure 4:**
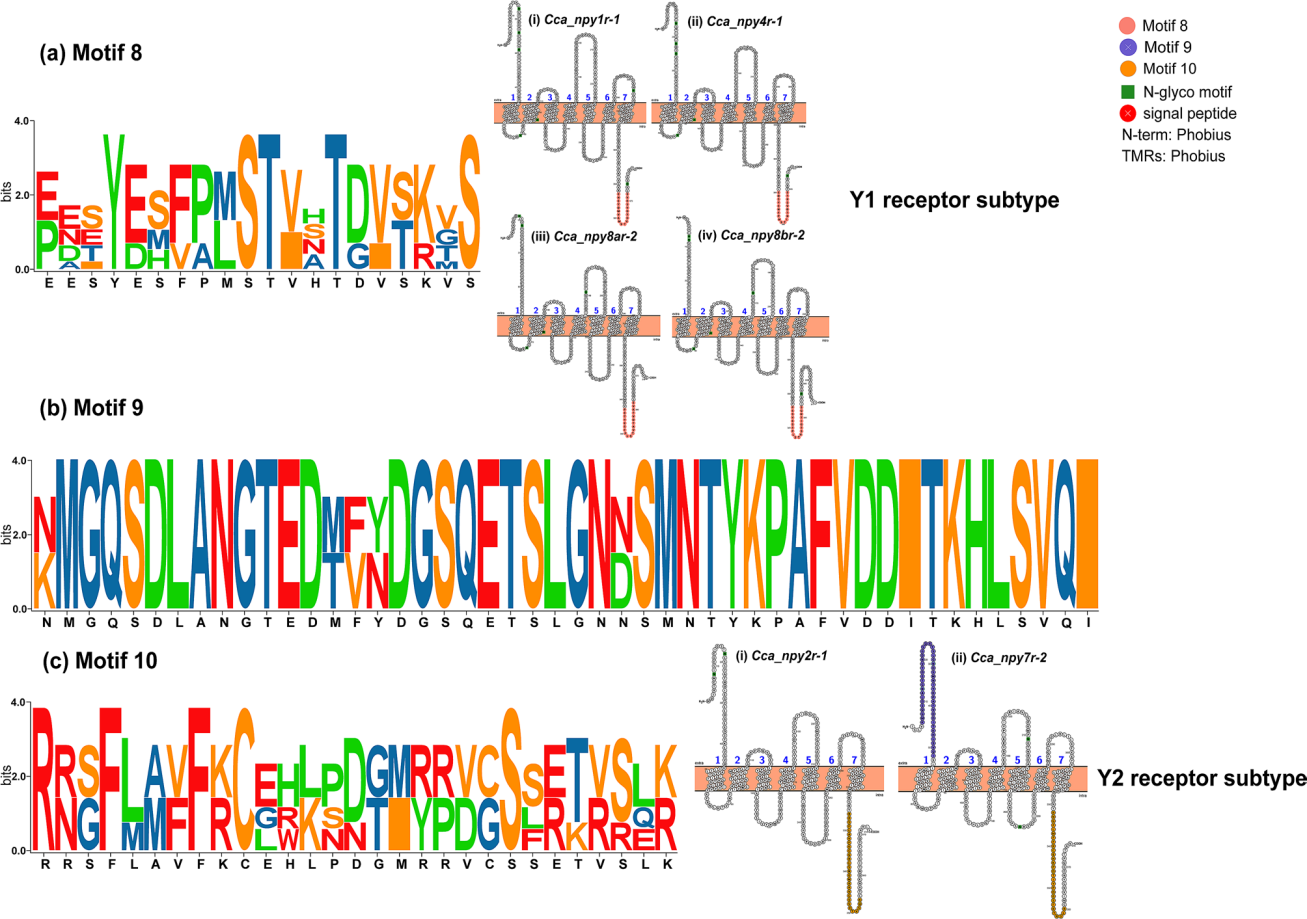
Structure and motif characteristics of two subtypes of NPY-family receptors in allotetraploid *C. carpio*. (**a**) Y1 subtype receptors in allotetraploid *C. carpio* and their common unique motif. Motif 8 is unique to the Y1 receptor subtype of *C. carpio*. On the left are a logo representation and distribution of motif 8 for the *C. carpio* NPY-family receptors. On the right are examples of four Y1 receptors (*Cca_npy1r-1*, *Cca_npy4r-1*, *Cca_npy8ar-2* and *Cca_npy8br-2*), showing the distribution of motif 8 in transmembrane domains. (**b**, **c**) Y2 subtype receptors in allotetraploid *C. carpio* and their common unique motifs. Motif 9 is specific to the N-terminal of Y2 subtype receptors, while motif 10 is unique to the C-terminal of Y2 subtype receptors. On the left are logos representing the distribution of motif 9 and motif 10 for the *C. carpio* NPY-family receptors. On the right are the transmembrane domains of an example of two Y2 receptors (*Cca_npy2r-1*, *Cca_npy7r-2*).

Nevertheless, we found that receptors of different subtypes have their unique motifs (Fig. 4). For Y1 subtype receptors, including *npy1r*, *npy4r*, *npy8ar*, *npy8br,* and their copies, there is a C-terminal specific motif 8. The logo distribution of motif 8 showed that the amino acid variants usually occur among different receptors rather than different copies of receptors (Fig. 4a). Furthermore, motif 9 is specific at the N-terminal of Y2 subtype receptors, while motif 10 is specific at the C-terminal, showing the structural differences between Y2 receptors (Fig. 4b,c).

### Chromosomal location and gene duplication of *npyr* genes

**Figure 5:**
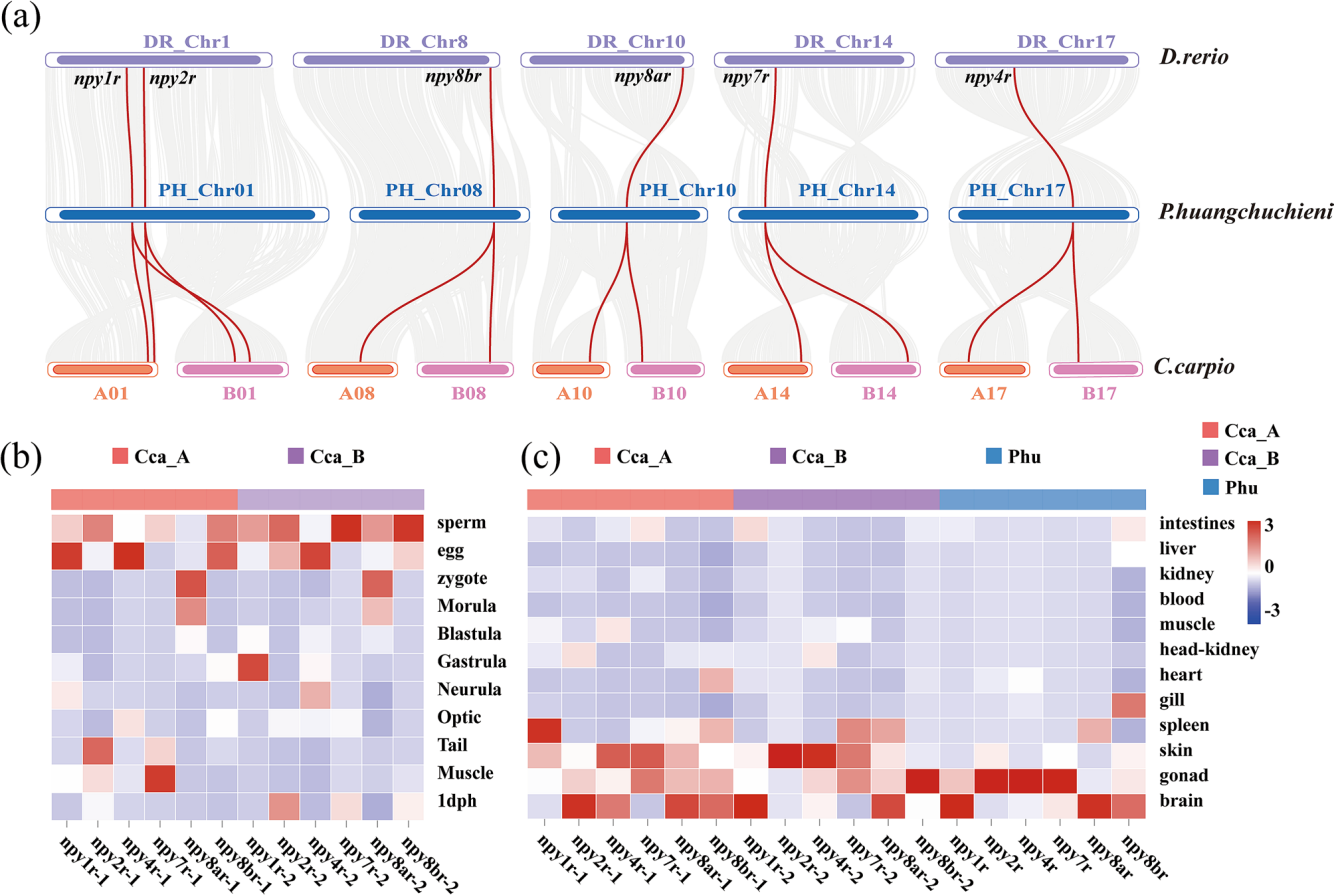
Chromosomal distribution and comparative expression profiles of NPY-family receptor genes from diploid reference species and subgenomes of allotetraploid *C. carpio*. (**a**) Chromosomal distribution and synteny plot of *npyrs* from three species of Cyprinidae. Gray lines indicate synteny blocks within diploid *D. rerio* and allotetraploid *C. carpio* and its B-subgenome progenitor-like diploid *P. huangchuchieni*, and red lines highlight the *npyr* gene pair. (**b**, **c**) The comparative expression profile between allotetraploid *C. carpio* subgenomes and its B-subgenome progenitor-like diploid *P. huangchuchieni.* The left heatmap shows subgenome comparative expression patterns of *npyr* genes during common carp embryo developmental stages, and the right heatmap displays the tissue-specific comparative expression profile of *npyr* genes among subgenomes of allotetraploid *C. carpio* and its B-subgenome progenitor-like diploid *P. huangchuchieni*, both with stage and tissue names listed on the right side of the panel, and all *npyr* gene names in *C. carpio* are listed at the bottom of the panel.

The allotetraploid *C. carpio* has two sets of subgenome A and B^23^. Comparing the *C. carpio* and its subgenome B progenitor-like diploid *P. huangchuchieni* with model species *D. rerio*, there were two copies of *C. carpio npyr* genes located on a pair of homologous chromosomes, which were derived from the two sets of subgenome A and B of the common carp genome, respectively (Fig. 5a). This implied that after the carp-specific fourth round of whole-genome duplication event (*Cs4R* WGD), *npyr* genes underwent a complete doubling and re-allocation of gene function in two subgenomes.

In addition, most *npyr* genes are evenly distributed among the ten paralogous chromosomes in *C. carpio* subgenomes, of which there are up to two *npyr* genes on chromosome A01 and B01. The six *npyr* genes of *C. carpio* subgenome B progenitor-like diploid *P. huangchuchieni* were located on five chromosomes, the same as in model species *D. rerio*, with *npy1r* and *npy2r* distributed on chromosome 1 together (Fig. 5a). The results showed that *npy1r* and *npy2r* in the three species of Cyprinidae are tandem duplication genes.

### Comparative expression patterns of *npyr* genes

In order to reveal the comprehensive expression patterns of *npyr* genes in allotetraploid common carp, we analyzed and compared the gene expression levels at different embryonic developmental stages in its subgenomes (Fig. 5b). The results showed that most genes were preferentially expressed in sperm and egg, except that two copies of *npy4r* were specifically expressed in the egg stage, and there was no such obvious biased subgenome expression of *npyr* genes at early developmental stages^41^. In addition, the specific expression genes in each developmental stage that appeared after fertilization, such as *npy8ar* genes, showed specific expression in fertilized eggs, and it was speculated that the Y8a receptor might be able to regulate key downstream pathways in the early stages of embryonic development. *Npy1r-2* and *npy7r-1* were specifically expressed during the gastrula and muscle contraction stages, respectively. In summary, the results showed that the global expression of *npyr* genes in sperm and egg gradually became the dominant expression of specific genes at specific stages of embryonic development in allotetraploid common carp, which is the dominant gene in the common carp reproductive system. This might hint that the role of NPY-family receptors has been gradually redeployed in regulating growth and immune function in individual developmental processes.

RT-PCR results for different tissues in *T. rubripes* showed that *npyr* genes are expressed in the brain and also expressed in other peripheral tissues^42^. In order to investigate the tissue-specific expression patterns of *npyr* genes in allotetraploid *C. carpio*, we analyzed transcriptome data from twelve tissues of *C. carpio* and *P. huangchuchieni*. It was found that most of the *npyr* genes were expressed in the brain, skin, and gonad (Fig. 5c). In the brain, *npy2r* and *npy4r* were highly expressed in *C. carpio* subgenome A, but low or no expression in *C. carpio* subgenome B and its progenitor-like diploid *P. huangchuchieni*, while *npy7r* was not expressed in the brains of all three. For other tissues, we found that *npy1r* of *C. carpio* subgenome A was exclusively expressed in the spleen, which demonstrated a related form of immunity to the immune system in the common carp. Most *npyr* genes apart from *npy8br* were commonly expressed in the skin of *C. carpio*, but a similar situation did not occur in *P. huangchuchieni*, which implied a homogeneity within *C. carpio* genomes. In addition, *npy8br* in *C. carpio* subgenome B was specifically highly expressed in the gonad, while *npy2r, npy4r,* and *npy7r* genes of *P. huangchuchieni* were highly expressed, which indicated that the tissue expression pattern of *C. carpio* subgenome B was more similar to that of *P. huangchuchieni*. In general, the above results all supported the role of *npyr* genes in the growth and immunity of organisms^43^. Similar gene expression patterns also provided evidence that *P. huangchuchieni* may be a possible progenitor of *C. carpio* subgenome B.

## Discussion

In this study, we investigated the NPY-family receptor genes of allotetraploid common carp and explored their evolutionary footprint and tissue distribution in different organisms. *S. salar*, *O. mykiss* and *C. auratus* who have experienced linage specific whole-genome duplications are thought to have led to the production of additional receptors and peptides in the NPY system^44^. We involved all the above species in this study, as well as the progenitor-like diploid species of allotetraploid *C. carpio*, such as *P. huangchuchieni* and *O. macrolepis*. The results showed that the number of *npyr* genes in tetraploid species was twice as much as that in diploid species. However, the distribution of the *npyr* genes in the autotetraploid and the diploid in the same habitat is not completely 2:1, which means that the gene loss may have occurred in *S. salar* and *O. mykiss* after the ancient whole-genome duplication event, but the gene copy produced by the recent polyploidy event in *C. carpio* and *C. auratus* has not changed much. This study elucidated the evolutionary fate of *npyr* genes after species-specific whole-genome duplication events in teleosts.

As is known, Y1 and Y5 receptors are usually considered as receptors for promoting food intake^10^. Taking Japanese medaka in marine habitat and zebrafish in freshwater habitat as model organisms, we found that Y1 receptor lost in teleost from marine habitat, but was detected in freshwater habitat species, and additional copies of Y1 receptor were found in goldfish, which may have experienced unique amplification^14^. In addition, a study on the expression profile of *npyr* genes in different tissues of common carp showed that Y1-1 was specifically expressed in the spleen^45^, suggesting the role of Y1 receptors in immunity^5,43^.

We found that the Y1 receptor was deficient in several fish in marine habitats but was present in all freshwater habitats. Whether the coincidence of distribution characteristics has anything to do with habitat remains unknown and needs further research to prove it. To explore the relationship between *npy1r* and the living environment of fish, we investigated the orthologues of *npy1r* in Ensembl, and a total of eleven fish had orthologues of this gene, of which ten were freshwater fish. Only Atlantic herring (*Clupea harengus*) was an exception. It lived in the Atlantic Ocean and was a type of marine migratory fish. The salinity range for a live environment is 5–35 ‰^46^. By constructing the species phylogenetic tree, we found that the genetic distance between this group and freshwater fish was closer, which might explain why, although it lived in seawater, it still had the *npy1r* gene, while other marine fish lacked this gene.

In addition, there were three copies of the Y1 receptor in goldfish, one more copy than in allotetraploid common carp, while there were only one copy of Y4 and Y7 in goldfish^14,47^. Since Y1 was the main feeding-promoting receptor, we believed that it might have additional functional differentiation and should be enhanced in goldfish. The *npyr* genes in common carp evenly distributed in two sets of subgenomes homologous to diploid species which confirmed the relatively new results of carp-specific polyploidy events^23^. The lack of copies of *npyr* genes in two ancient autotetraploid populations might be related to the early rediploidization^19^.

Because the Y5 receptor was not present in the teleost in this study, we suspected that this gene may have been lost in the evolution of teleost but retained in mammals and humans. Therefore, we investigated the orthologues of Y5 and found that they only existed in three ancient groups, *Scleropages formosus*, *Erpetoichthys calabaricus* and L*episosteus oculatus* which supported our conclusions. Up to now, Y1 and Y5 receptors have been ancient NPY receptor relics, which was confirmed in study^1,11^. Y5 existed in the early basal teleost but was lost in modern teleosts, whose genome became more complicated over time. In contrast, however, the third and fourth whole-genome duplication events from fish did not show any trace of the NPY5R gene.

Considering the allotetraploid background of *C. carpio*, the additional fourth round of whole-genome duplication events added to its genomic complexity. The gene copies generated through whole-genome duplication usually have various evolutionary fates, such as neofunctionalization, subfunctionalization, pseudogenization and gene loss, and functional conservation^48^. The evolutionary trend of the endocrine system of allotetraploid *C. carpio* after the fourth round of whole-genome duplication is an interesting topic, and existing research from our laboratory has found that the expression pattern of taste receptor genes indicated extensive gene functional differentiation^49^. The olfactory receptor gene family evolved asymmetrically between subgenomes, and the gene loss may have resulted in the reduction of the number and diversity of olfactory receptor genes^50^. Further in this study, our results showed that the *npyr* genes of allotetraploid common carp had an expressed preference among two subgenomes, and the B subgenome had a similar expression pattern with its diploid progenitor-like *P. huangchuchieni*. These studies provide insights into the evolutionary dynamics of the endocrine system of *C. carpio* after the fourth round of whole-genome duplication events.

## Conclusions

In conclusion, a comprehensive genomic identification of *npyr* genes in allotetraploid common carp and nine other representative species was conducted to display the landscape of *npyr* genes in vertebrates. Detailed information on the multiple alignments, phylogeny relationship, conservative motifs and structural analysis revealed two subtypes of twelve *npyr* genes in allotetraploid common carp. Gene tandem duplication and chromosomal location of *npyr* genes were performed via syntenic analysis to decipher the evolutionary process of allotetraploid common carp after the fourth round of whole-genome duplication events. Furthermore, the comparative expression profiles of embryonic development in allotetraploid common carp showed an expression preference between two subgenomes. In adult tissues, the expression pattern of common carp was similar to *C. carpio* subgenome B progenitor-like diploid P. *huangchuchieni*. Overall, a full genomic investigation and comparative expression profiles of *npyr* genes in allotetraploid *C. carpio* and related species could provide new insight into future exploration of the function of *npyr* genes and the neuroendocrine system of allotetraploid *C. carpio* and vertebrates.

## Supplementary Information

Supplementary Information 1.

Supplementary Information 2.

Supplementary Information 3.

Supplementary Information 4.
