# Supplementary material for: The neuropeptide Y receptor gene repository, phylogeny and comparative expression in allotetraploid common carp
Source: Sci Rep. 2022 Jun 8;12:9449. doi: 10.1038/s41598-022-13587-2 (PMC9177570; doi:10.1038/s41598-022-13587-2)
Supplement: Supplementary file 4 — Supplementary Information 4. [file 41598_2022_13587_MOESM4_ESM.pdf]

Supplementary materials:

Fig. S1 Secondary structure of *Cyprinus carpio* NPY receptor genes. each protein has characteristic seven-times transmembrane structure (7tm_1) and the amino acid sequence length vary from 370 to 381 with high sequence similarity.

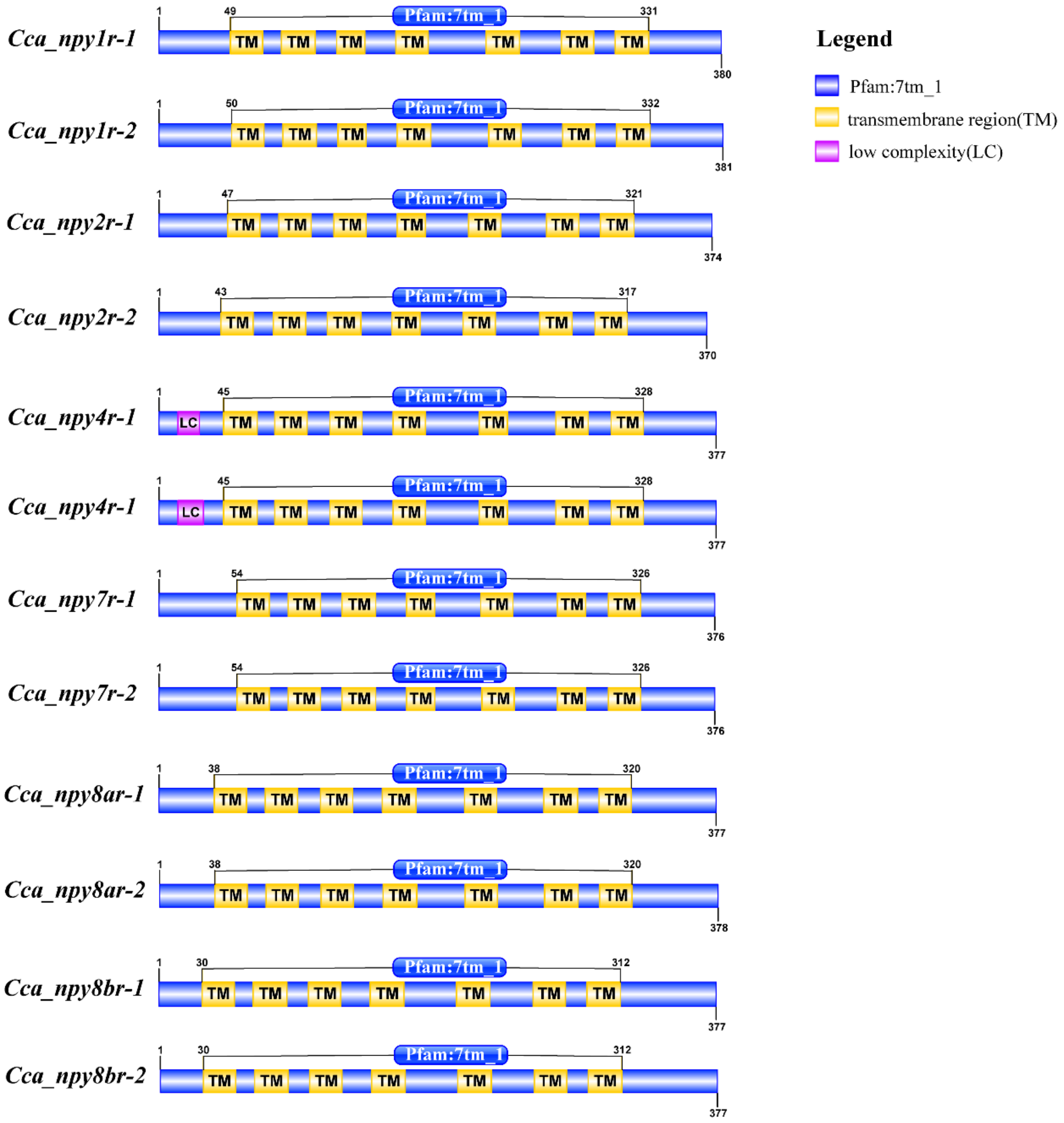

Supplementary File 1 Summary of all vertebrate protein sequences used in phylogenetic tree.

Supplementary File 2 Summary of all teleosts sequences used in selection pressure (dN/dS) analysis.

Supplementary File 3 Summary of npy2r amino acid sequences in all examined teleost.
